# People with lived experience of chronic conditions are key epistemic authorities in global health

**DOI:** 10.1371/journal.pgph.0005317

**Published:** 2025-10-10

**Authors:** Lavanya Vijayasingham, Mansi Chopra, Edith Mukantwari, Benny Prawira, Eman Shannan, Joab Wako, Maria Divina O'Brien, Mark Barone

**Affiliations:** 1 HealwithZeal Global CIC, London, United Kingdom; 2 Health Related Information Dissemination Amongst Youth (HRIDAY), New Delhi, India; 3 Africa Diabetes Alliance, Kampala, Uganda; 4 Into The Light Indonesia Suicide Prevention Community for Advocacy, Research and Education, Jakarta, Indonesia; 5 Aid and Hope Program for Cancer Patients Care, Gaza, Palestine; 6 Transplant Education Kenya, Nairobi, Kenya; 7 Mindwise Project and Focal Point Voices of SIDS (Small Island Developing States), San Fernando, Trinidad and Tobago,; 8 Intersectoral Forum of NCCs/NCDs in Brazil (ForumCCNTs), Sao Paulo, Brazil; PLOS: Public Library of Science, UNITED STATES OF AMERICA

The age of medical paternalism, where health professionals act as sole, well-meaning decision-makers for the health of populations, is over [1]. Of course, health professionals, researchers, and policymakers must remain epistemic authorities, especially in today’s era of pseudoscience and medical misinformation. However, this authority must be shared with those they serve, that is, those who are closest to and directly affected by the health problems that need solutions.

In the context of this article, the people they serve are us: people with lived experience (PWLE) of non-communicable diseases (NCDs), mental health, neurological, and all other chronic health conditions. When we are meaningfully engaged as lived experience experts, we provide health systems with real-time, real-world insights on our diverse, complex, and changing life needs, in our first-person voice. When we collaborate to co-design and co-produce health solutions through policies, programs, and research, we help health systems better meet the health needs of everyone. Together, we can build people-centred and learning health systems that work more efficiently, effectively, and equitably. Together, we can equip populations to be healthier, more resilient, and, in turn, more economically and socially productive.

## What does meaningful engagement mean?

The WHO Framework defines meaningful engagement of people living with NCDs, mental health and neurological conditions as the *“respectful, dignified, and equitable inclusion of individuals with lived experience in processes and activities, within enabling environments where power is transferred, valuing lived experience as expertise, and applying it to improve health outcomes*” [2,3]. This framework calls for implementation through sustainable financing, power-sharing, stigma reduction, integrated approaches, capacity-building, and institutionalised mechanisms; emphasising principles of dignity, equity, transparency, inclusivity, institutionalisation, and context sensitivity in its processes, quality and impact [2,3].

In practice, meaningful engagement is much more than our visible representation on advisory boards or document authorship lists. In research, this can be lived experience experts serving as research co-investigators, rather than just data collectors, or organising community access and engagement. In policy and health systems governance, this can manifest as lived experience experts having genuine relationships, power, ability, and influence in decision-making, policy co-creation or reform processes, where they guide implementation and hold systems accountable. For example, in Japan, a lived-experience-led Dementia Working Group formed strong working ties with policymakers, leading to the co-development of national dementia policies, community strategies, and awareness initiatives [4]. Recent high-level commitments such as the Bridgetown Declaration in 2023 and the 4th International Conference on Small Island Developing States Declaration in 2024 signal the institutionalisation of meaningful engagement of people living with NCDs & mental health conditions, shared decision-making and formalised participatory approaches within health system governance and policy processes.

## Engagement still falls short

Meaningful engagement is not yet mainstream. It remains inconsistently implemented across regions and disease areas. Too often, the terms are dictated by institutions, leaving us, lived experience experts, with limited scope to influence decisions, processes or outcomes. Gaps in funding, power, and access to information constrain our participation. Many of us are still positioned as passive subjects in research, or are invited to events for symbolic inclusion, and tasked with emotive storytelling to ‘humanise’ meetings. At its worst, engagement can sometimes feel like a public relations tactic or ‘ethics-washing’.

With faith that global health can do better, lived experience experts from around the world advocated for the integration of meaningful engagement into the post-2025 NCD and mental health agenda [5]. In solidarity with this call, we draw from our personal stories of living with chronic conditions and the barriers we face in advocating for change to outline why and how global health must engage more meaningfully with us as lived experience experts.

## Meaningful engagement is a human right that repairs epistemic injustice

We all have the right not only to healthcare but also to participate in shaping it; a principle affirmed by the 1978 Alma-Ata Declaration [6] and reinforced by the 2024 WHO resolution on social participation [7]. Health decision-making expertise is not confined to doctors or researchers from affluent institutions. Calls to decolonise knowledge demand that outdated hierarchies shaped by a narrow medical perspective, colonialism, elite saviourism and global north dominance be dismantled [8]. True co-production must extend beyond symbolism or performative tick-boxing to address the long-standing legacy of epistemic injustice towards people with lived experience. True collaboration is measured by the extent to which our embodied knowledge, shaped by survival and struggle, is valued and put into action in global health.

## Meaningful engagement strengthens health systems

Investments in meaningful engagement can have a multiplier effect in health systems, similar to vaccines: preventing harm and fostering collective benefits, even in low-resource settings. Working formatively with expert insights from our lived experiences can reduce costly complications, avoid ineffective interventions, and bolster resilience in times of crisis. This requires sustainable financing that ensures fair pay, adequate skills, and logistics for the expert work we do [9]. Without sustainable financing, engaging with us is extraction, not empowerment.

## Meaningful engagement acknowledges the diversity of experience and pluralistic perspectives

No single story captures the diverse realities of chronic conditions across contexts and over time. Our chronic health conditions are too often framed only through trauma, disruption, stigma, and loss, but with the right support, we can also transform crisis into meaningful lives of wellness, happiness, and purpose [10]. Yet, our resilience is inequitably resourced: intersectional identities and histories, access to healthcare, employment and income security, social relationships or community support, and structural protections such as anti-discrimination laws shape whether our health conditions deepen disadvantage or are buffered by care resources [11]. Meaningful engagement must cohesively reflect the diversity of access and experience, while also embracing the complex and at times contradictory cultural, social, and systemic worldviews that shape our lives.

## Meaningful engagement creates safe spaces

Publicly disclosing our lived experiences can sometimes threaten our careers, reputations, relationships, safety, and families. This is particularly true when these records are used to discriminately exclude us from jobs and private health insurance coverage. Stigma persists all around the world, and privacy is often prioritised. Despite these personal costs and trade-offs, some of us choose visibility to change unjust systems, while others wisely prioritise privacy to protect themselves. To expand the pool of diverse lived experience experts who engage with health systems, we need safe spaces, stronger legal protections, and cultural shifts that remove these engagement barriers.

## Conclusion

By adopting and implementing the 2025 UN Political Declaration on NCDs and mental health at national levels, member countries can recognise the unique expertise of people living with, or caring for their family members with chronic conditions; that is, the value we bring to health systems when we co-shape policies and programmes across the care continuum. This presents a positive opportunity for all countries to leverage meaningful engagement as a strategic approach to developing strong, high-quality, responsive, and resilient health systems that are more equitable and efficient. Each step towards valuing our diverse voices, investing in our safe and supported participation, and embedding co-production into policy, research, and practice through sustainable resources is meaningful for us. When done well and consistently around the world, meaningful engagement can move us all closer towards achieving ‘health for all.’
